# The anti-tumor function of the IKK inhibitor PS1145 and high levels of p65 and KLF4 are associated with the drug resistance in nasopharyngeal carcinoma cells

**DOI:** 10.1038/s41598-019-48590-7

**Published:** 2019-08-19

**Authors:** Hong Lok Lung, Rebecca Kan, Wai Yin Chau, On Ying Man, Nai Ki Mak, Chun Hung Fong, Wai Ho Shuen, Sai Wah Tsao, Maria Li Lung

**Affiliations:** 1 Department of Biology, Hong Kong Baptist University, Kowloon Tong, Hong Kong (SAR) P.R. China; 2 Department of Clinical Oncology, University of Hong Kong, Pokfulam, Hong Kong (SAR) P.R. China; 3 School of Biomedical Sciences, Li Ka Shing Faculty of Medicine, The University of Hong Kong, Pok Fu Lam, Hong Kong P.R. China; 4 Center for Nasopharyngeal Carcinoma Research, Li Ka Shing Faculty of Medicine, University of Hong Kong, Pok Fu Lam, Hong Kong (SAR) P.R. China; 5 Present Address: Ketchum Pte. Ltd., 30 Merchant Road, Riverside Point, #03-12, Singapore, Singapore; 6 Present Address: Division of Medical Oncology, National Cancer Centre Singapore, Singapore, Singapore

**Keywords:** Targeted therapies, Apoptosis

## Abstract

We and others have previously shown that the canonical nuclear factor kappa-B (NF-κB) pathway is essential to nasopharyngeal carcinoma (NPC) tumor development and angiogenesis, suggesting that the NF-κB pathway, including its upstream modulators and downstream effectors, are potential therapeutic targets for NPC. The inhibitor of upstream IκB kinase (IKK), PS1145, is a small molecule which can specifically inhibit the IκB phosphorylation and degradation and the subsequent nuclear translocation of NF-κB. The present study aims to determine the anti-tumor activity of PS1145 on NPC. Our results showed that PS1145 significantly inhibited the growth of tumorigenic NPC cell lines, but not in the normal nasopharyngeal epithelial cell line. Results in the *in vivo* study showed that low concentration of PS1145 (3 mg/kg) could significantly suppress the subcutaneous tumor formation in the nude mice bearing NPC xenografts. Apparent adverse effects were not observed in the animal study. Drug resistance against PS1145 seems to be associated with the increased levels of active NF-kB p65 and change of expression levels of kruppel-like factor 4. As can be seen, PS1145 appears to be a safe agent for animal experiments and its effects are tumor-specific, and the proteins associated with the drug resistance of PS1145 are implied.

## Introduction

Nasopharyngeal carcinoma (NPC) arises from the epithelium of the nasopharynx and is characterized by the inflammatory tumor microenvironment with copious amount of lymphocyte infiltration^1^. Chronic Epstein-Barr virus (EBV) infection, dietary and other environmental factors, and genetics are believed to play a crucial role in NPC development. Radiotherapy with or without chemotherapy has been the standard treatment for NPC^2^. However, cancer cells develop resistance to the conventional anti-cancer drug and radiation treatments for tumors in a high percentage of NPC patients, which results in tumor recurrence and distant metastasis. These unfavorable events are the major causes of NPC death. Together with the undesirable side-effects from conventional anti-cancer therapies, development of a more specific targeted therapy for NPC is necessary^2^, but that remains a major challenge in the field due to the lack of understanding of this unique cancer type.

The presence of activated nuclear factor kappa-B (NF-κB) has established a causal link between inflammation and cancer. It has now become clear that NF-κB plays a critical role in cancer development and progression^3^. The active NF-κB complex is a homo- or heterodimer composed of proteins from the NF-κB/Rel family: NF-κB1 (p50), NF-κB2 (p52), RelA (p65), RelB, and c-Rel. In the canonical/classical pathway, before cell stimulation most p50/p65 dimers reside in the cytoplasm in a complex with the inhibitor protein, inhibitor of nuclear factor kappa-B (IκB). The degradation of IκB proteins is initiated by the signal-induced phosphorylation of IκB, which is executed by the IκB kinase (IKK) complex. The released p50/p65 dimers enter the nucleus and initiate transcriptional activities associated with inflammation.

Cumulative evidence has indicated the intimate relationship of NF-κB and NPC carcinogenesis. A number of studies has demonstrated the frequent over-expression or activation of NF-κB in NPC cell lines and tissues^4–7^. The NF-κB signaling cascade can be activated by the EBV latent infection in nasopharyngeal epithelial cells^5^. In addition to the alteration by EBV infection, genetic alterations of several NF-κB regulators including *TRAF2*, *TRAF3*, *NFKBIA*, *TNFAIP3*/*A20*, *LTBR*, and *CYLD1* also contribute to the aberrant NF-κB activation in NPC^6,8–10^. Several inflammatory cytokines such as TNF-α and IL-1 are well-known potent inducers of NF-κB and are reported to be elevated in the NPC tumors. This suggests that the NF-κB activation could be directly induced by these inflammatory cytokines in the tumors^1^.

Our previously identified NPC tumor suppressor genes, Cysteine-Rich Intestinal Protein 2 (CRIP2)^11^ and Transforming Growth Factor-beta Binding Protein 2 (LTBP2)^12^, were shown to inhibit the tumor formation by suppressing the canonical NF-κB p65-induced pro-angiogenic and epithelial–mesenchymal transition (EMT) activities. These results indicate the importance of the NF-κB pathway in tumor formation, angiogenesis, and invasion in NPC^11^. In addition, loss- and gain-of-function analyses of p65 were performed to demonstrate its direct functional roles in *in vivo* tumor growth, colony formation ability, tumor-associated angiogenesis, EMT, cell proliferation, and cell migration/invasion in NPC cells^7,12^. As can be seen, the canonical NF-κB pathway is essential to tumor development as well as angiogenesis in NPC, suggesting that the NF-κB pathway including its upstream modulators and downstream effectors, is a potential therapeutic target for NPC.

**Figure 1:**
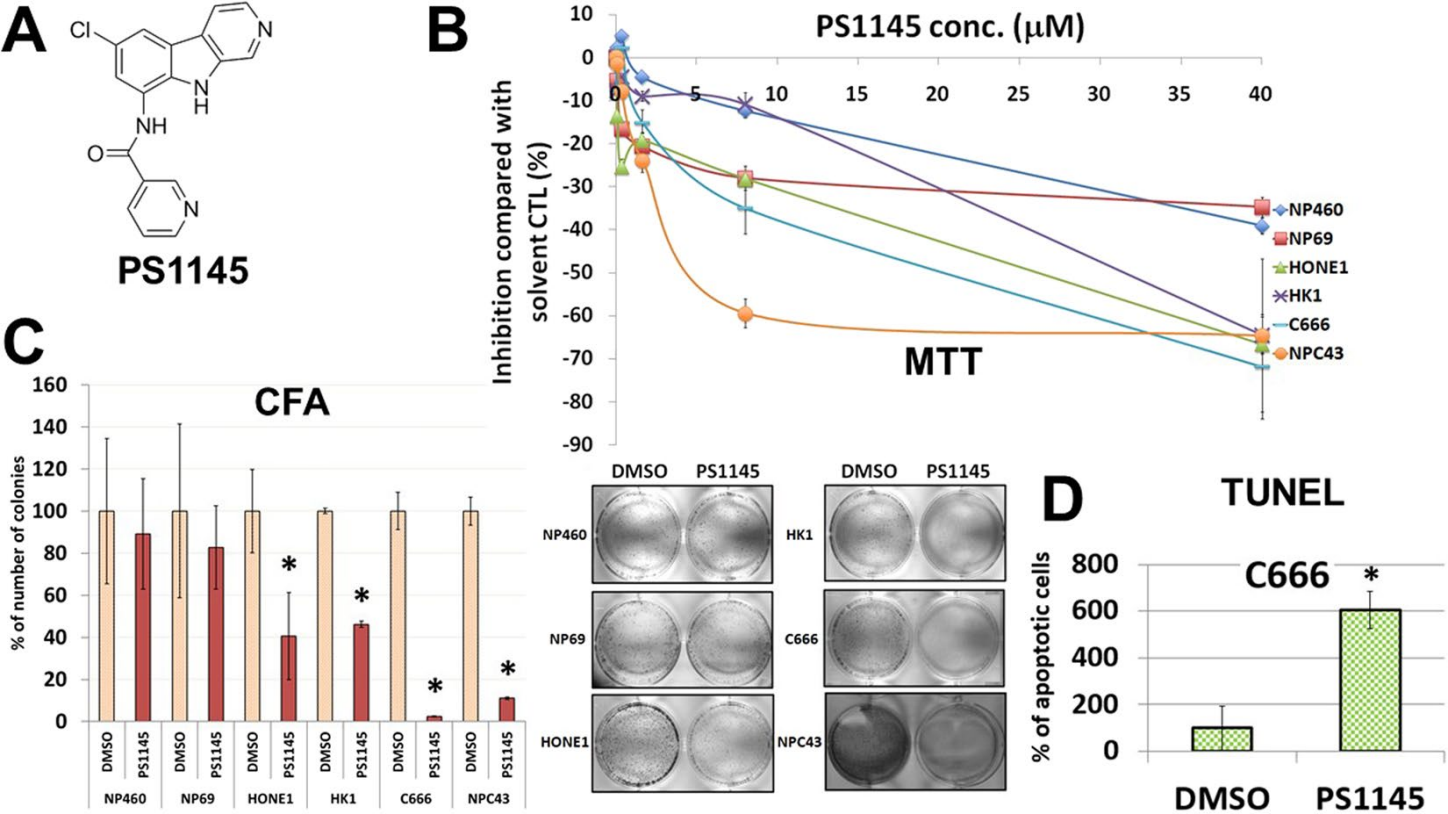
(**A**) Structure of PS1145. (**B**) Effects of the small molecule kinase inhibitor PS1145 on cell viability in NPC and NP cell lines. The cell viability for all NPC cell lines were determined using the MTT assay (on day 3 after the treatment). (**C**) 2D CFA analysis of the effects of PS1145 on the colony-forming abilities of the selected NPC and NP cell lines. The bar chart shows the percentage of colony formation by each cell line treated with DMSO solvent control and PS1145. These experiments were conducted in triplicates. *p value < 0.05. Representative images of the CFA results are shown. (**D**) Apoptosis analysis of the effects of PS1145 in C666 cells treated. The TUNEL assay was used to detect the apoptotic cells on day 3 after the treatment with 32 μM PS1145. The bar chart shows the percentage of number of apoptotic cells formed after treatment with PS1145 or the solvent control (DMSO). *p value < 0.05.

There are several different pharmacological strategies to target NF-κB. They include repression of the DNA binding activities of NF-κB, stabilization of IκB inhibitors by proteasome inhibitors, and inhibition of upstream IKKs^13^. The understanding of the unique properties of IKKβ among other serine-threonine kinases contributes to successful development of specific IKKβ inhibitors^14^. Of these, the small molecule PS1145 (Fig. 1A), derived from a β-carboline natural product^15,16^, has been extensively evaluated in various *in vitro* assays by different groups^13^. With an IC_50_ in the nanomolar range, PS1145 can effectively inhibit the IKK complex, abrogate IκB phosphorylation and degradation and the subsequent activation of NF-κB^15,16^, and blocks the TNF-α release in lipopolysaccharide (a well-known stimulus of NF-κB) treated mice^13,14^. The therapeutic role of PS1145 in carcinogenesis was initially demonstrated in multiple myeloma, as PS1145 could inhibit the pro-inflammatory cytokine production and cell proliferation^16^.

To our knowledge, there are very few pre-clinical studies targeting the NF-κB pathway as a therapeutic target in NPC. In the present study, we proposed to use a panel of NPC cell lines to study the effects of PS1145 on the status of NF-κB activities, tumor cell growth, induction of apoptosis, and *in vivo* tumor formation in NPC. In this study, we aimed to determine whether: (1) the IKK inhibitor PS1145 had the potential to be used as an anti-cancer drug to suppress the primary tumor of NPC, and (2) NPC cells could acquire resistance in gene(s) and pathway(s) associated with the drug resistance in the long-run after the PS1145 treatments.

## Results

### Effects of PS1145 on *in vitro* NPC cell growth

In order to accomplish the first objective, we investigated the effects of PS1145 on cell growth and tumor formation. *In vitro* cell growth was detected by the routine 3-(4,5-dimethylthiazol-2-yl)-2,5-diphenyltetrazolium bromide (MTT) cell viability assay and the colony formation assay (CFA) for a panel of NPC cell lines (including HONE1, HK1, and the EBV-positive C666 and NPC43). Two immortalized nasopharyngeal (NP) epithelial NP460 and NP69 cell lines were used as non-tumorigenic controls for comparison with the tumorigenic cell lines, to demonstrate the specificity of PS1145 in induction of tumor cell death (Fig. 1B). From the results of the MTT assay, the IC_50_ values of HONE1, HK1, C666, and NPC43 in response to PS1145 were 26.5, 25.8, 8.7, and 6.7 μM, whereas the IC_50_ values of NP460 and NP69 were» 40 and 37.2 μM. The 2D CFA was performed for the NPC cell lines treated either with DMSO solvent control or 32 μM PS1145. The colony-forming abilities of HONE1, HK1, C666, and NPC43 were significantly suppressed with PS1145 (Fig. 1C). However, NP460 and NP69 remained largely unaffected by this drug, which indicates that this drug specifically inhibits NPC cells, and not the immortalized epithelial cells (Fig. 1C).

### Effects of PS1145 on NPC cell growth and tumor formation and downstream signaling pathways

**Figure 2:**
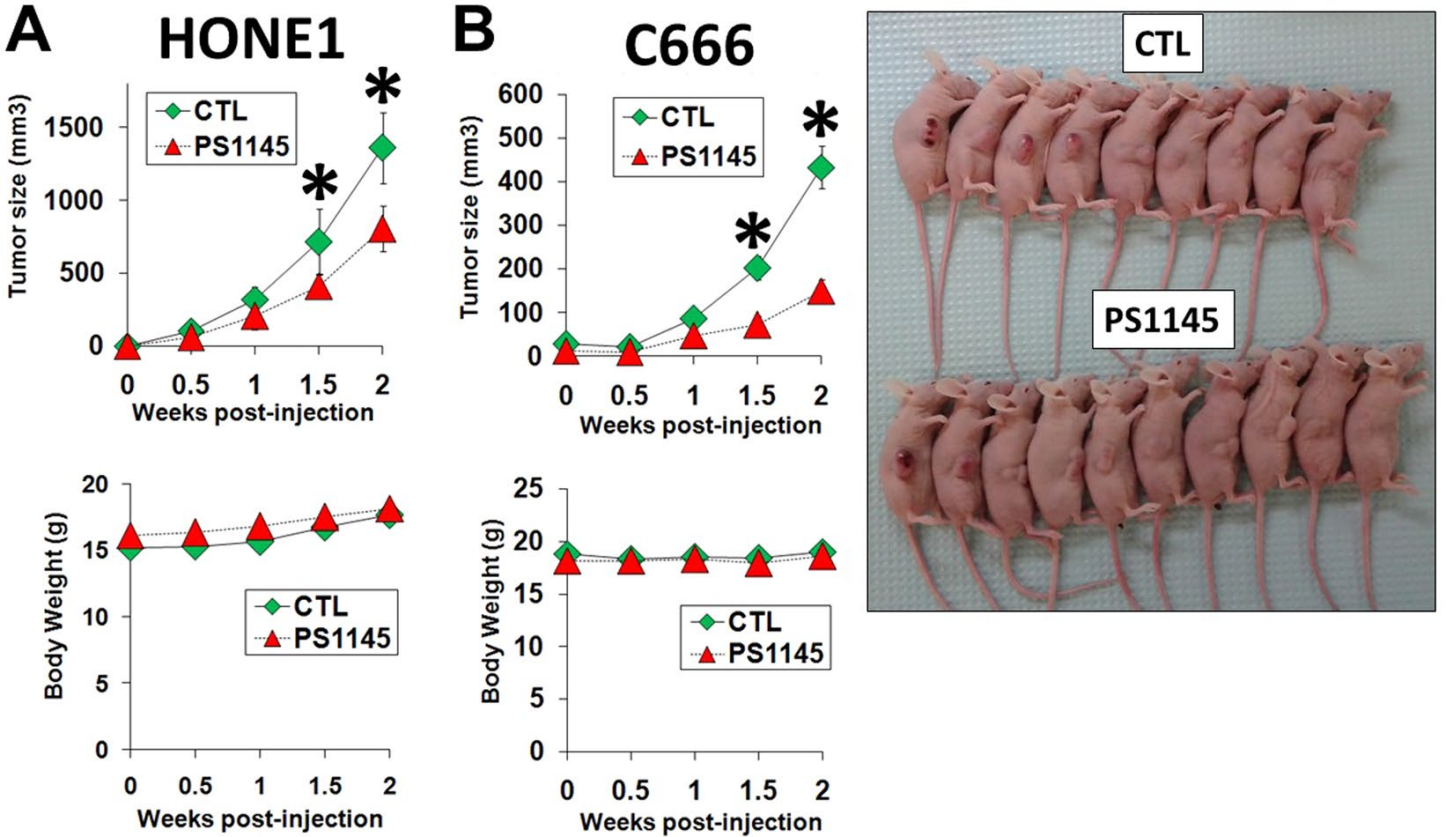
*In vivo* studies of the effects of PS1145 treatment on NPC cell lines (**A**) HONE1 and (**B**) C666. The cells were subcutaneously grafted on the right flank of nude mice. A total of 24 nude mice were used per cell line, with each group consisting of 12 mice. The mice with either treated with solvent control or PS1145 (3 mg/kg). The drug was administered bi-weekly via intravenous tail-vein injection. The average tumor sizes (upper panel) and average body weights (lower panel) of the animals were measured weekly. *p value < 0.05. Photos of tumor-bearing mice (injected with C666 cells) taken at the end of experiment.

In order to understand the mechanism for how PS1145 could inhibit the NPC cell growth, apoptosis was assessed by the terminal deoxynucleotidyl transferase-mediated dUTP nick end labelling (TUNEL) assay. The 32 μM PS1145 treatment was shown to induce apoptosis in the selected C666 cells (Fig. 1D); this EBV-positive cell line was chosen for the apoptosis analysis because it was most sensitive to PS1145. The PS1145-induced apoptosis could be also observed in the other two NPC cell lines (Supplementary Fig. S1). To further support this finding in *in vivo* models, HONE1 or C666 cells were injected subcutaneously into the right flanks of 24 nude mice. The mice were then subdivided into two groups (12 mice per group), to form a control and a treatment group. The mice in the treatment group for both HONE1 and C666 fared better than the control group, with significantly smaller tumor burdens with a low dose of PS1145 (3 mg/kg) (Fig. 2). Overall, C666 was more responsive to the inhibitory effects of PS1145 than the HONE1, as shown by an earlier suppression of tumor growth, and a significantly smaller average tumor size throughout the study (Fig. 2). Importantly, from the gross appearance and body weight of the animal, there was no apparent adverse effect for mice treated with PS1145 (Fig. 2).

**Figure 3:**
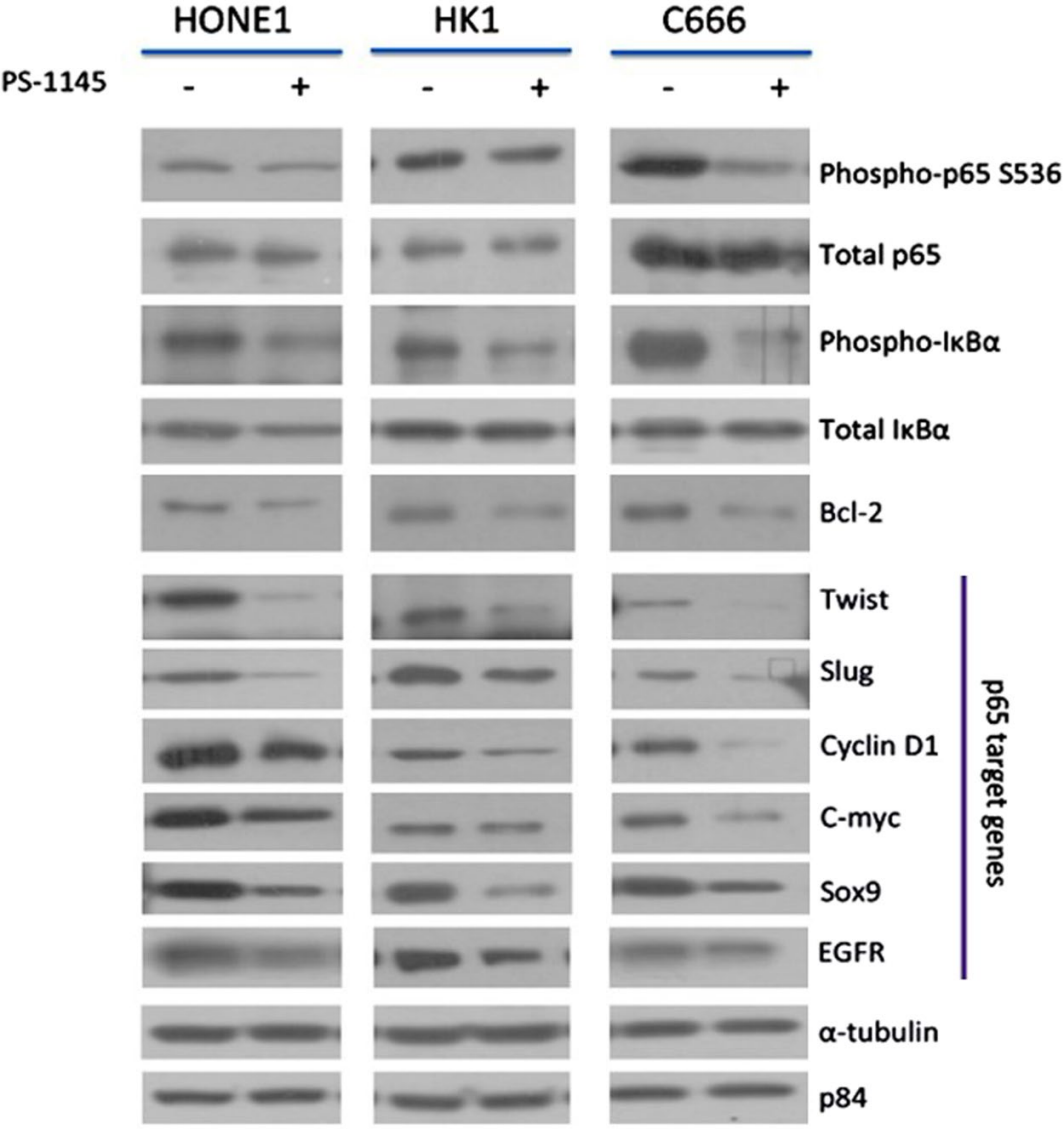
The effects of PS1145 (32 μM) on the NF-κB biochemical signaling pathway of NPC cell lines (HONE1, HK1, and C666). The protein expression of the canonical NF-κB pathway upstream and downstream target proteins in response to the PS1145 treatment of the NPC cells was detected by Western blot analysis.

The HONE1, HK1, and C666 cell lines after treatment with PS1145 were analyzed for their biochemical signaling activities using Western blot analysis. The biochemical results across all three cell lines were consistent. The PS1145 treatments led to a consequential down-regulation of phosphorylated p65 and IκBα (Fig. 3). Since the proteins that were directly involved in the NF-κB pathway were inhibited, the downstream p65-related target genes were also analyzed. These genes are known to harbor the NF-κB binding sites at their promoter regions, hence, allowing NF-κB to initiate their gene transcription. Evidently, protein levels of the anti-apoptotic protein, Bcl-2, was decreased (Fig. 3). Other proteins that showed similar down-regulated trends were transcription factors, twist, slug, c-myc, and sox9. Cyclin D1, a cell-cycle regulator protein, and EGFR, were also markedly inhibited (Fig. 3). These results confirmed that the PS1145 elicits an effective NF-κB p65 inhibitory response in NPC cell lines that could translate into both functional and biochemical consequences.

### Establishment of PS1145-resistant cell lines

**Figure 4:**
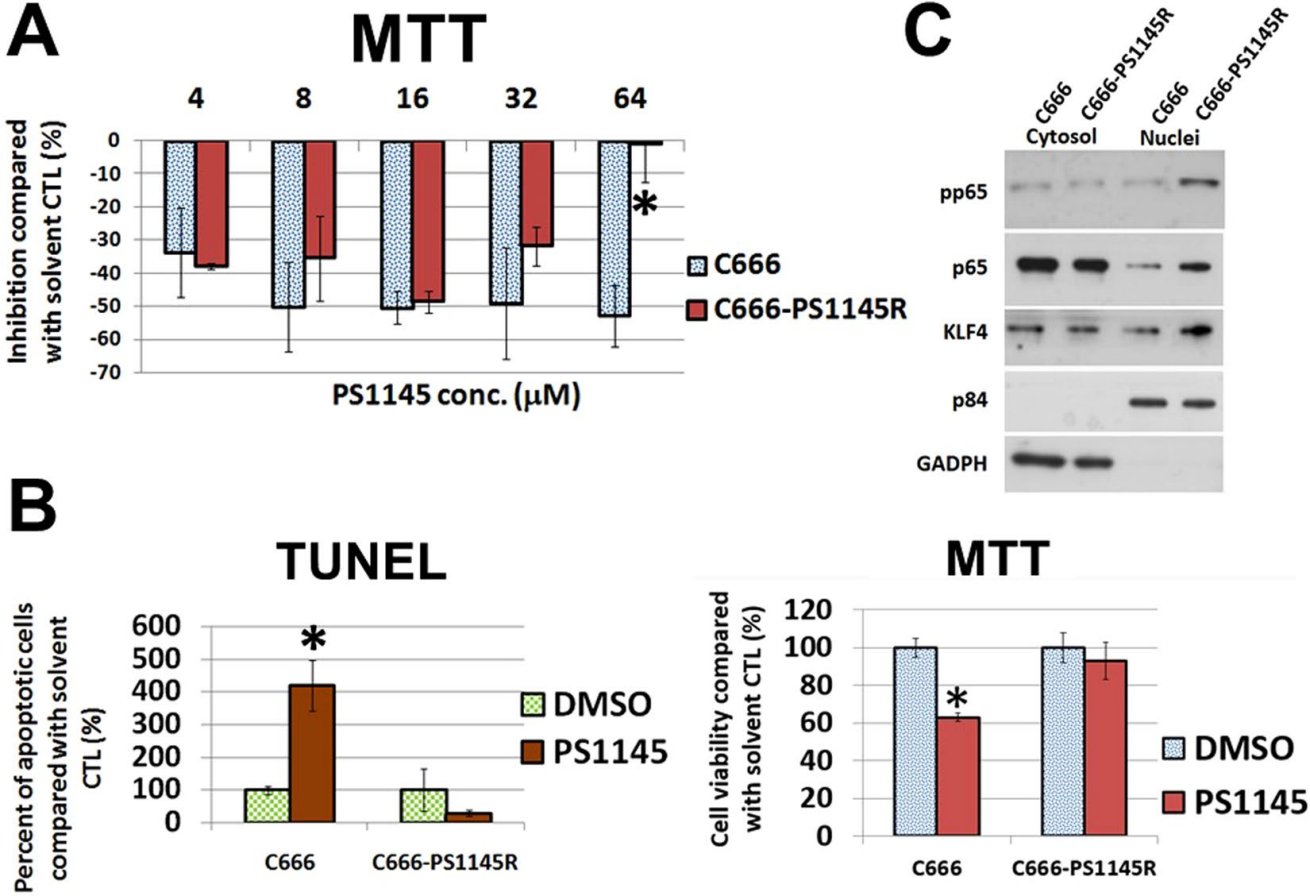
(**A**) Comparison of cell viability of C666 cells and its resistant derivative C666-PS1145R in response to PS1145 (4–64 μM). The cell viability was determined by MTT assay on day 3 after the treatment. The bar chart shows the percentage of number of viable cells treated with different concentrations of PS1145. *p value < 0.05. (**B**) Apoptosis analysis of the effects of PS1145 in C666 cells and its resistant cell line C666-PS1145R. The effects of 32 μM PS1145 on the formation of apoptotic cells of the parental C666 and its derivative C666-PS1145R were detected by TUNEL assay on day 3 after the treatment. The bar chart shows the percentage of number of apoptotic cells formed in the treatment with PS1145 or DMSO. *p value < 0.05. A parallel MTT assay was performed for the same batch of cell lines. (**C**) Western blot analysis of protein expression in C666 versus C666-PS1145 cells. The cytosolic and nuclei fractions of the proteins of interest were compared between the two cell lines. The p84 and GADPH are the nuclei and cytosol loading controls.

We planned to establish PS1145-resistant cell lines from *in vitro* cell culture. In brief, the CFA was used and the NPC cell lines were exposed to PS1145 until only a few surviving clones appeared; these colonies were expanded in the absence of PS1145. An incremental increase of PS1145 dose was applied to the surviving cells with repeating on and off cycles of PS1145. The MTT assay was used to confirm the acquisition of drug resistance compared with the parental cell lines. Four NPC cell lines including HONE1, HK1, C666, and NPC43 cells were originally involved for the establishment of the drug resistant cell clones. However, the PS1145 dose response of the cell clones derived from HONE1, HK1, and NPC43 were found to be nearly similar to their parental cell lines, as reflected by the MTT assay (data not shown). Fortunately, a PS1145-resistant C666 cell line (C666-PS1145R) was obtained (Fig. 4A). The response to low dose PS1145 (4–16 μM) was similar to the parental C666, but at high does (32 & 64 μM) the cell viability was much lower; the number of viable cells of C666-PS1145R in response to 64 μM PS1145 was almost the same as the solvent control, while 50% decrease was observed in the parental cell line (Fig. 4A). As apoptosis was shown to be one mechanism responsible for the PS1145 toxicity in C666 cells in the previous section (Fig. 1C), to further confirm C666-PS1145R had acquired resistance against PS1145, the TUNEL assay was performed; results show that there was no increase in number of apoptotic cells when C666-PS1145R was treated with 32 μM PS1145, whereas there was a more than 4-fold increase in apoptotic cell numbers of the parental C666 (Fig. 4B). A MTT assay was performed in parallel for these two C666 cell lines to confirm the change of viable cell numbers (Fig. 4B).

### Investigation of gene(s) and signaling pathway(s) related to drug resistance to PS1145 in NPC cells

**Figure 5:**
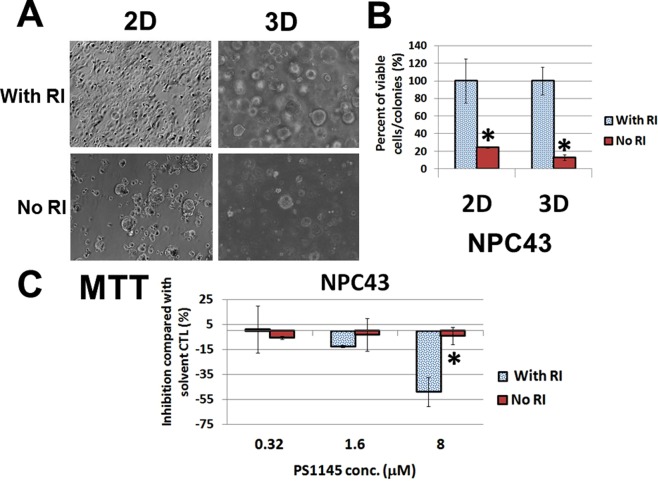
(**A**) Morphological difference of NPC43 cells cultured with or without the Rock inhibitor (RI) Y27632 in 2D and 3D culture conditions. (**B**) Percentage of number of viable NPC43 cells/colonies in 2D/3D culture in the absence of RI compared with in the presence of RI (4 μM). Numbers of viable cells were determined by MTT assay on day 3 after the treatment. (**C**) Comparison of number of viable cells of NPC43 cells treated with or without RI (4 μM) in response to PS1145 (32 μM). Numbers of viable cells were determined by MTT assay on day 3.

**Figure 6:**
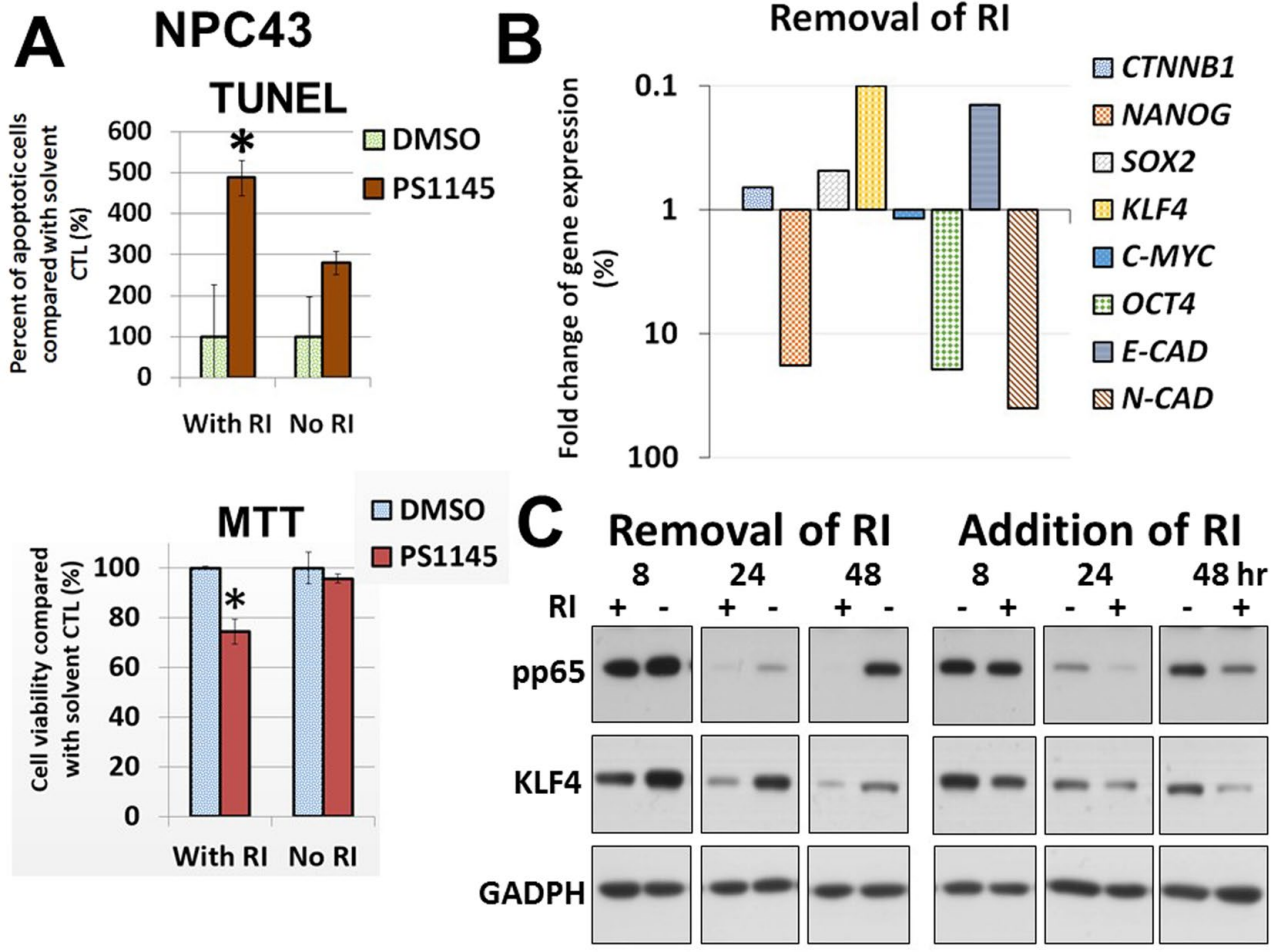
(**A**) Apoptosis analysis of the effects of PS1145 in NPC43 cells in the presence or absence of the Rock inhibitor (RI) Y27632. The effects of 4 μM RI in response to 32 μM PS1145 on the formation of apoptotic cells were detected by TUNEL assay on day 3 after the treatment. The bar chart shows the percentage of number of apoptotic cells formed by the treatment with PS1145 in the presence or absence of Y27632. A parallel MTT assay was performed for the same batch of cell lines. (**B**) Gene expression analysis of stemness and differentiation genes in NPC43 cells cultured with or without 4 μM Rock inhibitor (RI) Y27632. (**C**) Western blot analysis of the protein expression of phospho-p65 (pp65) and KLF4 after removal or addition of the RI (4 μM) in NPC43 cells is shown. The treatment time is indicated. Both long and short exposure versions for detection of pp65 are shown in Fig. S6, and only the long exposure is shown in this figure. The differences of pp65 expression at 8 hours is more obvious in the short exposure version.

**Figure 7:**
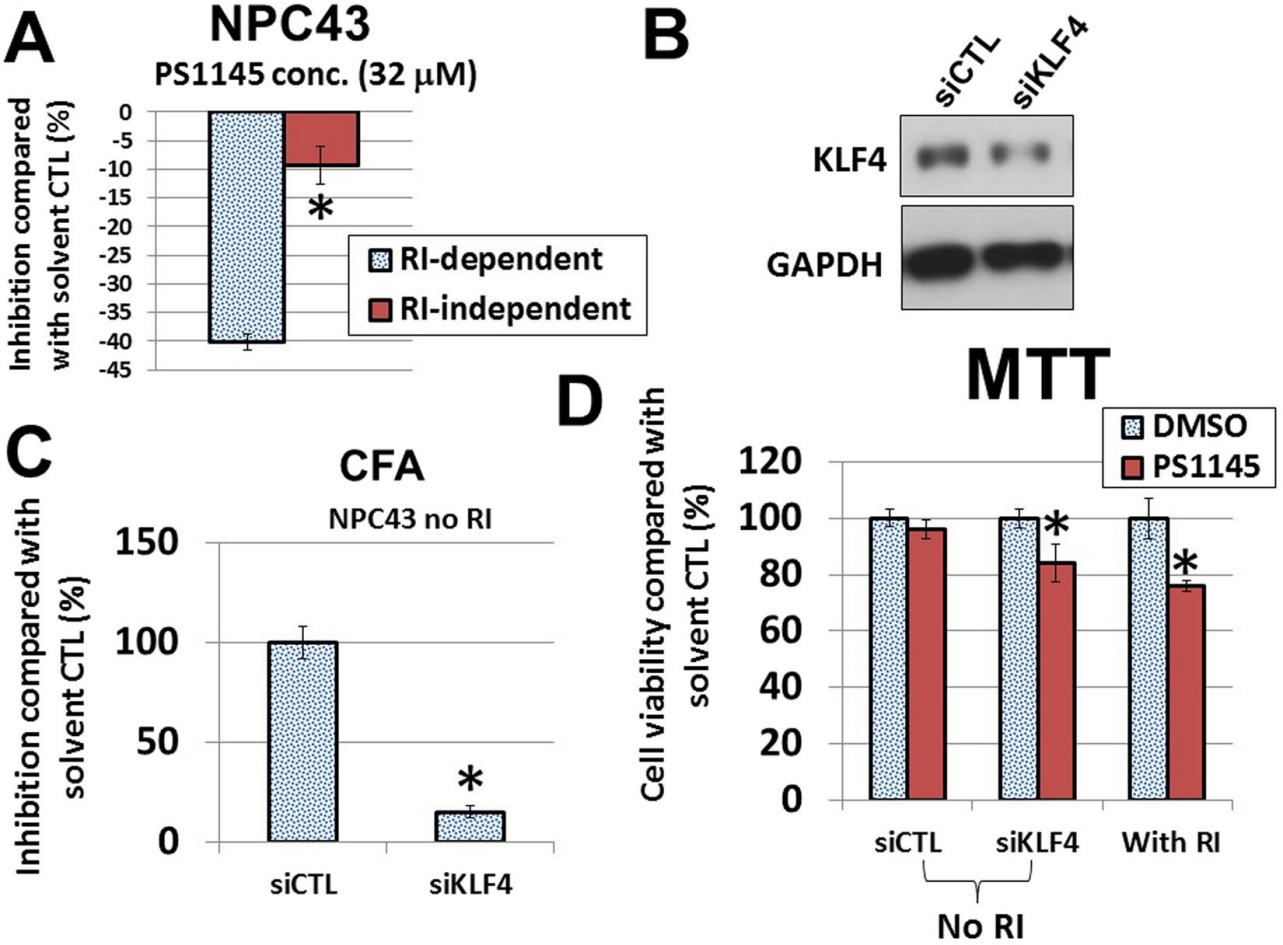
(**A**) Comparison of cell viability of the RI-dependent versus the RI-independent NPC43 cells in response to PS1145 (32 μM). The cell viability was determined by MTT assay on day 3 after the treatment. *p value < 0.05. (**B**) Western blot analysis of the protein expression of KLF4 after siRNA knockdown in NPC43 cells. siCTL, scramble control siRNA. (**C**) CFA analysis of the effects of *KLF4* siRNA on the colony-forming abilities of NPC43 cells. These experiments were conducted in triplicates. *p value < 0.05. (**D**) Cell viability in response to PS1145 (16 μM) after treatment with *KLF4* siRNA in NPC43 cells (in the absence of RI). MTT assay was performed to detect the cell viability. *p value < 0.05.

As aforementioned, the long-term drug treatment could not generate a PS1145-resistant phenotype for NPC43 cells. This particular cell line is normally cultured with the Rock inhibitor (RI) Y27632, which is a well-known agent for culturing stem cells^17^. Removal of this RI could significantly reduce the cell growth in both 2D and 3D culture conditions by 5- to 10-fold (Fig. 5A,B). The presence of the RI could induce both the cell proliferation and colony formation, but some cells survived in the absence of Y27632 (Fig. 5A). Interestingly, these surviving cells seem to be associated with the PS1145 resistance, as removal of Y27632 resulted in dramatic reduction of cytotoxicity to PS1145 (Fig. 5C). There was no obvious change in viable cell numbers, when NPC43 cells were treated with the three concentrations of PS1145 without the addition of Y27632 in the culture medium, whereas 1.6 and 8 μM PS1145 could reduce the viable cell numbers to ~10 and 50%, respectively, when Y27632 was present (Fig. 5C). Furthermore, there was no significant change in the numbers of apoptotic cells after the PS1145 treatment when culturing without the RI, while a 5-fold increase of cell death was observed when RI was added (Fig. 6A). A parallel MTT assay was performed for these two culture conditions to confirm the change of viable cell numbers (Fig. 6A). It is likely that the surviving NPC43 cells without the addition of RI are PS1145-resistant, and removal of RI can be a screening method for the PS1145-resistant NPC43 cells. As can be seen, this type of drug resistance might be related to the stemness properties of the NPC cells. The qPCR analysis was performed to compare the gene expression of some major stemness, differentiation, and epithelial-mesenchymal transition (EMT) rmarkers in the presence and the absence of the RI. Around 10-fold and 7-fold increase of *KLF4* and *ECAD(CDH1)* were detected, respectively, when RI was removed from the culture medium; in contrast ~18-fold, 19-fold, and 40-fold decrease of *NANOG*, *OCT4*, and *NCAD (CDH2)* was observed, respectively (Fig. 6B). To further confirm the p65 activity and KLF4 expression are associated with PS1145-resistance, the PS1145-resistant C666-PS1145R cell line (Fig. 4A) was used to check these candidate proteins. The Western blot results show that the expression levels of the nuclear p65 and its active phosphorylated form and the nuclear KLF4 were much higher in the resistant C666-PS1145R cells (Fig. 4C). The gene expression results in NPC43 were also confirmed with the Western blot analysis (Fig. 6C). As can be seen, after removal of RI, the KLF4 expression was consistently increased from 8 to 48 hrs. When RI was added to the culture medium, the KLF4 expression was reduced from 8 hr to 48 hrs (Fig. 6C). Interestingly, the phospho-p65 activity was also induced, when RI was removed, and the addition of RI resulted in a reduction of the p65 activity (Fig. 6C). It seems that the increase in p65 activity and KLF4 expression are associated with the the resistant cells of NPC43, when RI was absent in the culture. In agreement with this observation, the late passge (>160 passage) of this cell line had become RI-independent for cell growth,and had acquired the PS1145 resistance property (Fig. 7A). However, the addition of RI to C666-PS1145R could not enhance its sensitivity to PS1145 (Supplementary Fig. S3). It seems that the RI could not directly contribute to the sensitization of NPC cells to PS1145. This implies the elevation of the canonical NF-κB pathway in a subpopulation of the two cell lines might play some roles in maintaining the NPC cell survival, when exposed to high dose of PS1145.

To obtain functional evidence of KLF4 in the PS1145 resistance, we reduced the KLF4 expression levels in NPC43 and C666 cells with siRNA (Fig. 7B; Supplementary Fig. S2). The colony formation ability of NPC43 was significantly decreased by more than 6-fold (Fig. 7C). Interestingly, the *KLF4*-knockdown cells were more sensitive to PS1145 in the absence of RI and the decrease was comparable to the presence of RI (Fig. 7D). A similar result was observed in the C666-PS1145R cells, when *KLF4* was inhibited (Supplementary Fig. S2), although this result was not as obvious as that of NPC43. These results suggested that KLF4 is one of the factors that is likely to contribute to the PS1145 resistance in NPC cells.

## Discussion

In this study, we aimed to investigate whether PS1145, a small molecule IKK inhibitor, could be used as a targeted anti-tumor agent and/or deemed as an appropriate adjuvant drug that fits into the current conventional therapy. We clearly show the efficacy of PS1145 in *in vitro* and *in vivo* NPC studies, especially in the EBV-positive C666 and NPC43 cell lines. Apoptosis is one of the mechanisms for PS1145 to inhibit the NPC cell growth of these two cell lines. The high sensitivity of C666 and NPC43 cells to PS1145 might suggest that inhibition of the NF-κB pathway could be equally effective in suppressing the actual NPC tumors with prevalent EBV-infection. Indeed, administration of PS1145 to nude mice inoculated with NPC cell lines was performed and the results show that a low concentration of 3 mg/kg PS1145 could significantly suppress the subcutaneous tumor formation in both C666 and HONE1 cell lines. The effects of PS1145 on C666 *in vivo* and *in vitro* tumor growth are more obvious than for other NPC cell lines; this may be due to its highest total and active p65 protein levels (unpublished observation). For the body weights of the animals, there was no significant change in the presence of the IKK inhibitor during the whole experimental periods for the two independent experiments. PS1145 appears to be safe for animal experiments and its effects are tumor-specific.

Based on the comparison of protein and gene expression in the C666-resistant and its parental cell, the high expression levels of active p65 could be one of the factors responsible for better survival at high concentrations of PS1145. On the other hand, the NPC43 resistant model was generated when the culture condition was altered. Without the addition of the ROCK Inhibitor (RI) Y27632 (for both short and long term) could acquire resistance against the toxicity of PS1145 in this EBV-positive NPC cell line; it is likely that a drug resistant subpopulation of NPC43 cells was selected in the absence of RI. Y27632 is known to be a selective inhibitor of the Rho associated kinase p160-ROCK. This RI is used to culture human embryonic stem cells and human induced pluripotent stem cells, as treatment with Y27632 avoids dissociation-induced apoptosis of these stem cells, increasing the survival rate and maintaining pluripotency during sub-cultivation and thawing of these cells^17,18^. When comparing the gene expression of culture conditions with and without the RI, about 10-fold alteration of gene expression of *KLF4*, *ECAD*, *NANOG*, *OCT4*, and *NCAD* was observed. The Western blot results confirmed that the KLF4 protein expression was inversely related to the presence of RI. The stemness/differentiation reprogramming seems to be important for NPC43 cells to acquire the drug resistance. In contrast, the addition of RI to the C666-PS1145R cells induced no significant change, as the growth of the parental C666 cells is not dependent on the presence of RI and differs to NPC43 cells. The effect of RI on the sensitivity to PS1145 is cell context dependent. In addition, the acquisition of drug resistance was associated with the increase of active p65 levels in these EBV-positive cells. Interestingly, increase of both KLF4 protein expression and phospho-p65 levels was also detected in the C666 resistant cell lines. The functional assay results of the *KLF4* knockdown have indicated that this stem cell factor could play a role in the PS1145 resistance in NPC cells. Taken together, it is likely that this drug resistance property was due to a subpopulation of NPC cells with high NF-κB activities; *KLF4* could be one of the key genes to enable the NPC cells to survive against the toxicity of PS1145. Expression patterns of p65 and KLF4 might be useful to predict the drug resistance against PS1145 and probably other anti-tumor agents in NPC patients.

In general, NF-κB plays a critical role in blocking apoptosis and, thus, allows tumor cells to escape from cell death induced by chemo- or radiotherapy. For example, resistance of human cervical carcinoma cells to cisplatin is partly mediated via enhancement of cisplatin-induced NF-κB activation. Indeed, inhibition of the NF-κB activities has been shown to increase the sensitivity of tumor cells to ionizing radiation and chemotherapy drugs^19,20^. Interestingly, KLF4 has been reported as a direct target of the NF-kappaB/p65, as the Toll-like Receptor 9 (TLR9) signaling was shown to be essential for propagation and self-renewal characteristics of prostate cancer cells, and could induce the transcription factors NF-kappaB/RELA and STAT3 to co-regulate KLF4 and NKX3.1 gene expression by directly binding to their promoters^21^. KLF4 has been shown to be associated with chemotherapy resistance in a few tumors including head and neck, liver, and bone cancers^22–24^. In head and neck squamous cell carcinoma, persistent KLF4 expression is associated with a poor disease-specific survival. The correlation is even stronger in patients with advanced disease; the high levels of KLF4 expression could significantly enhance multi-drug resistance, *in vitro* migration/invasion potential, and *in vivo* tumor formation^22^. KLF4 was also found to mediate cisplatin resistance in the hepatocellular carcinoma stem-like cells by upregulating expression of gamma-glutamylcysteine synthetase and the subsequent glutathione synthesis^23^. In osteosarcoma cells, the KLF4 expression was significantly increased in response to various anticancer drugs and can regulate drug resistance by at least partially upregulating it direct target gene *high-mobility group box 1*^24^.

By inhibiting the NF-κB/p65 pathway with PS1145, the detrimental effects seen in the natural EBV-positive cell lines clearly show a dependency on this signaling route. These findings suggest that the EBV-positive NPC cell lines were more sensitive to the inhibition of the canonical NF-κB pathway, which indicates that the drug could potentially be useful for clinically diagnosed EBV-positive NPC tumors. The low dose PS1145 is safe in our animal studies and could reduce the primary tumor sizes derived from various NPC cell lines. Interestingly, the present study had shed some light on which genes/proteins are associated with the drug resistance in NPC, and high level expression of p65 and its active form is one of those resistant markers. Development of more potent agents against the NF-κB/p65 signaling pathway is required to eradicate the drug resistant cells. Some of these factors seem to be universal for drug resistance derived from other anti-cancer agents. These drug resistance-associated genes could help to predict tumor recurrence after NPC therapies.

## Methods

### Cell lines

Culture conditions for the NPC cell lines were as described^25^. The immortalized nasopharyngeal epithelial cell lines NP460^26^ and NP69^27^ were cultured as described. The newly derived NPC43 cells were maintained in RPMI1640 with 10% serum and 4 μM ROCK inhibitor (RI) Y27632 (Enzo Life Sciences). All cell lines used in the current study were confirmed to be mycoplasma-negative and have been genotype authenticated.

### IKK inhibitor PS1145

Five millimole PS1145 dihydrochloride (Sigma-Aldrich, St Louis, MO, USA) stock was prepared and was dissolved in dimethyl sulfoxide (DMSO). For *in vivo* models, 3 mg/kg PS1145 (5 mM stock was diluted 10× in sterile water) for use per injection, while the solvent control consisted of 10% DMSO in sterile water. The drug was administered bi-weekly via intravenous tail-vein injection.

### MTT 3-(4,5-dimethylthiazol-2-yl)-2,5-diphenyltetrazolium bromide assay

The MTT assay was performed as previously described^25^. In brief, 4 × 10^3^ to 1.2 × 10^4^ cells were seeded into 96-well plates and incubated for about 3–7 days, depending on the experimental criteria. A volume of 30 μl of MTT (Sigma-Aldrich, St. Louis, MO, USA) solution (5 mg/ml) was added to the cells and further incubated for 1.5 hours. The purple precipitate was then dissolved with DMSO (Sigma-Aldrich, St. Louis, MO, USA) before measuring its absorbance at 490 nm with the Multiskan FC microplate photometer (Thermo Scientific Inc., Beverly, MA, USA). The half maximal effective/inhibitory concentration (IC_50_) of an inhibitor/antagonist is a measure of the effectiveness of the drug in inducing half of the maximum biological response. The quantitative index of PS1145 for each cell line was determined using the MTT assay to plot a dose-response curve that was arbitrarily based on the antagonistic effect of increasing concentrations of PS1145 on cell survival.

### 2-Dimensional (2D) colony formation assay

In 2D colony formation assay, NPC cells were seeded and cultured at low density on 6-well plates. Cells were then fixed with 10% formalin (Sigma-Aldrich, St. Louis, MO, USA) and stained with 1× diluted Giemsa reagent (Sigma-Aldrich, St. Louis, MO, USA). The numbers of colonies were quantitated with the Gel Doc XR system (Bio-Rad Laboratories, Berkeley, CA, USA).

### 3-Dimensional (3D) tumor sphere formation assay

The 3D tumor sphere formation assay was performed as previously described^28^. Cells were seeded in low cell density (2 × 10^3^ cells/well) on a 24-well ultralow attachment plate (Corning, Acton, MA) as mentioned.

### TUNEL assay

The TUNEL assay was performed by utilizing an *In situ* cell death detection kit (Roche Diagnostics, Basel, Switzerland), as described^25^.

### qPCR analysis

Semi-quantitative and quantitative PCR were performed as reported^25^. The primers used in this study were described in our previous publication^12^.

### Western blot analysis

Cell lysate preparation and Western blot analysis was performed and the antibodies were used as reported^12^. The raw data are provided in the Supplementary Figs S4 to S9.

### Tumorigenicity assay

Subcutaneous injection was performed as previously described^25^. Briefly 1 × 10^7^ cells were administered subcutaneously into the flanks of 6 to 8 weeks old female BALB/cAnN-nu (nude) mice. The tumor mass was measured on a weekly basis and its growth kinetics was plotted accordingly. If visible tumor formations were observed, representative tumors were excised and reconstituted into cell cultures for subsequent molecular analyses. The raw data are provided in the Supplementary Tables S1 to S12.

### Cell transfection with siRNA

C666-1 or NPC43 cells were transfected with small interference-RNA (siRNA) (50 nM) complexed with Lipofectamine 2000 (Invitrogen, CA, USA) for 72 hours according to manufacturer’s instruction. For NPC43, 4 μM RI was necessary to be included in the transfection mixture. Transfected cells were harvested and subjected to the following assays. Silencer Pre-designed human *KLF4* siRNA (Ambion, MA, USA) was used in parallel with Silencer negative control siRNA (Ambion).

### Statistical analysis

The statistical analyses were performed as described in our previous study^29^. All *in vitro* assay results represented the arithmetic mean ± standard error of triplicate determinations. The standard error of mean (SEM) was used to calculate the standard error of the *in vivo* assay. The Student’s *t* test was used to determine the confidence levels for group comparisons. A *p* value < 0.05 was considered as statistically significant.

We confirm that all methods were performed in accordance with the relevant guidelines and regulations

### Ethics approval and consent to participate

All the animal experiments were approved by the Committee on the Use of Live Animals in Teaching and Research of The University of Hong Kong.

## Supplementary information

Supple Figs & Tables
