# Supplementary material for: The anti-tumor function of the IKK inhibitor PS1145 and high levels of p65 and KLF4 are associated with the drug resistance in nasopharyngeal carcinoma cells
Source: Sci Rep. 2019 Aug 19;9:12064. doi: 10.1038/s41598-019-48590-7 (PMC6700134; doi:10.1038/s41598-019-48590-7)
Supplement: Supplementary file 1 — Supple Figs & Tables [file 41598_2019_48590_MOESM1_ESM.pdf]

Supplementary data

Title: The anti-tumor function of the IKK inhibitor PS1145 and high levels of p65 and KLF4 are associated with the drug resistance in nasopharyngeal carcinoma cells

Hong Lok Lung[1#*], Rebecca Kan[2,a#], Wai Yin Chau[1], On Ying Man[1], Nai Ki Mak[1,4], Chun Hung Fong[1,b], Wai Ho Shuen[2,c], Sai Wah Tsao[3,4], Maria Li Lung[2,4*]

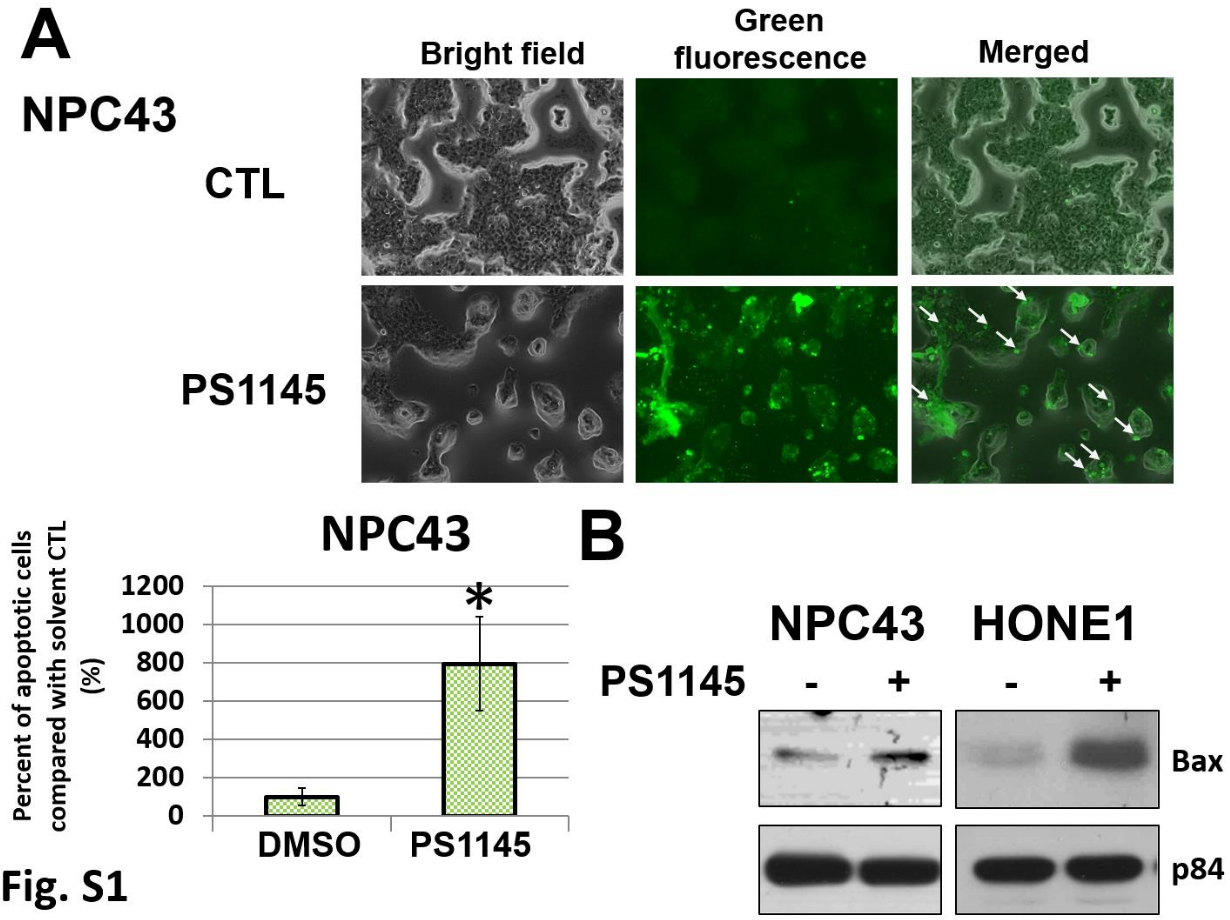

Fig. S1

Figure S1. (A) Apoptosis analysis of the effects of PS1145 in NPC43 cells. The TUNEL assay was used to detect the apoptotic cells on day 3 after the treatment with 32 μM PS1145. Representative results are shown in the upper panel. The white arrows indicate the apoptotic cells. The bar chart shows the percentage of number of apoptotic cells formed after treatment with PS1145 or the solvent control (DMSO). *, p value < 0.05. (B) Western blot analysis of the protein expression of the apoptotic factor Bax after the PS1145 treatment (32 μM).

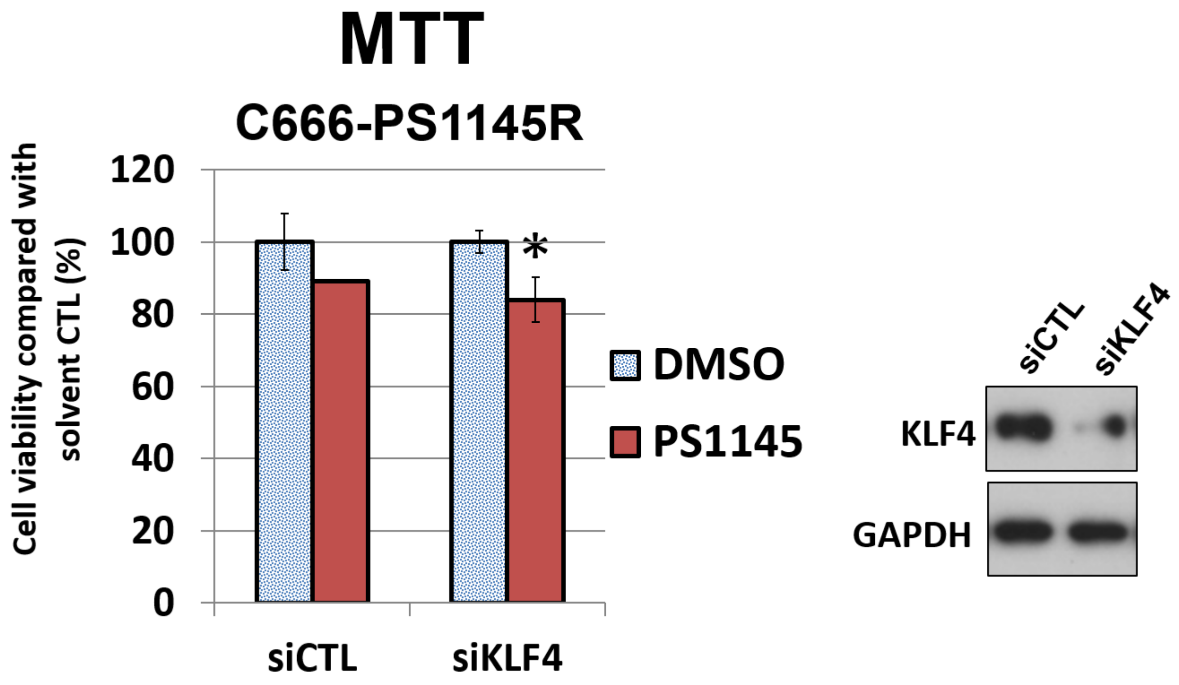

Fig. S2

Figure S2. Cell viability in response to PS1145 (32 μM) after treatment with *KLF4* siRNA in C666-PS1145R cells. The MTT assay was performed to detect the cell viability. Western blot analysis of the protein expression of KLF4 after siRNA knockdown in these cells. siCTL, scramble control siRNA.

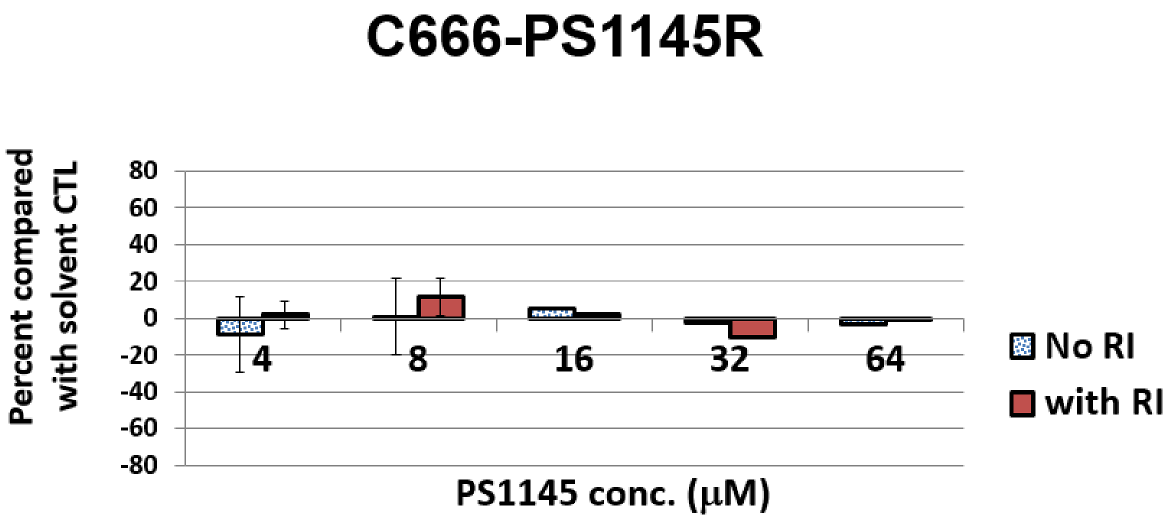

Fig. S3

Figure S3. Examination of the effects of the ROCK inhibitor (RI) Y27632 on the cell viability of C666-PS1145R in the presence or absence or various amounts of PS1145. The cell viability was detected by MTT.

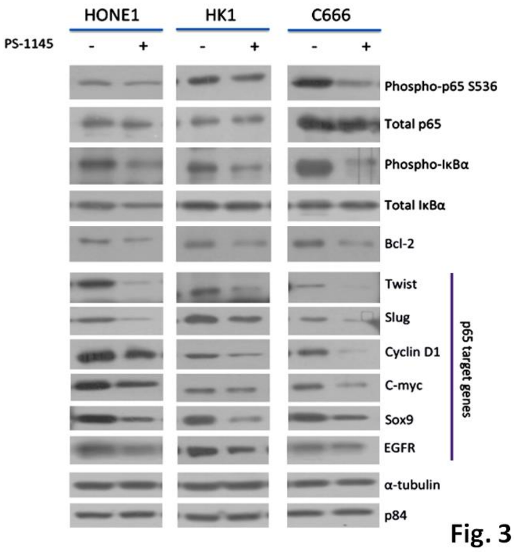

Fig. 3

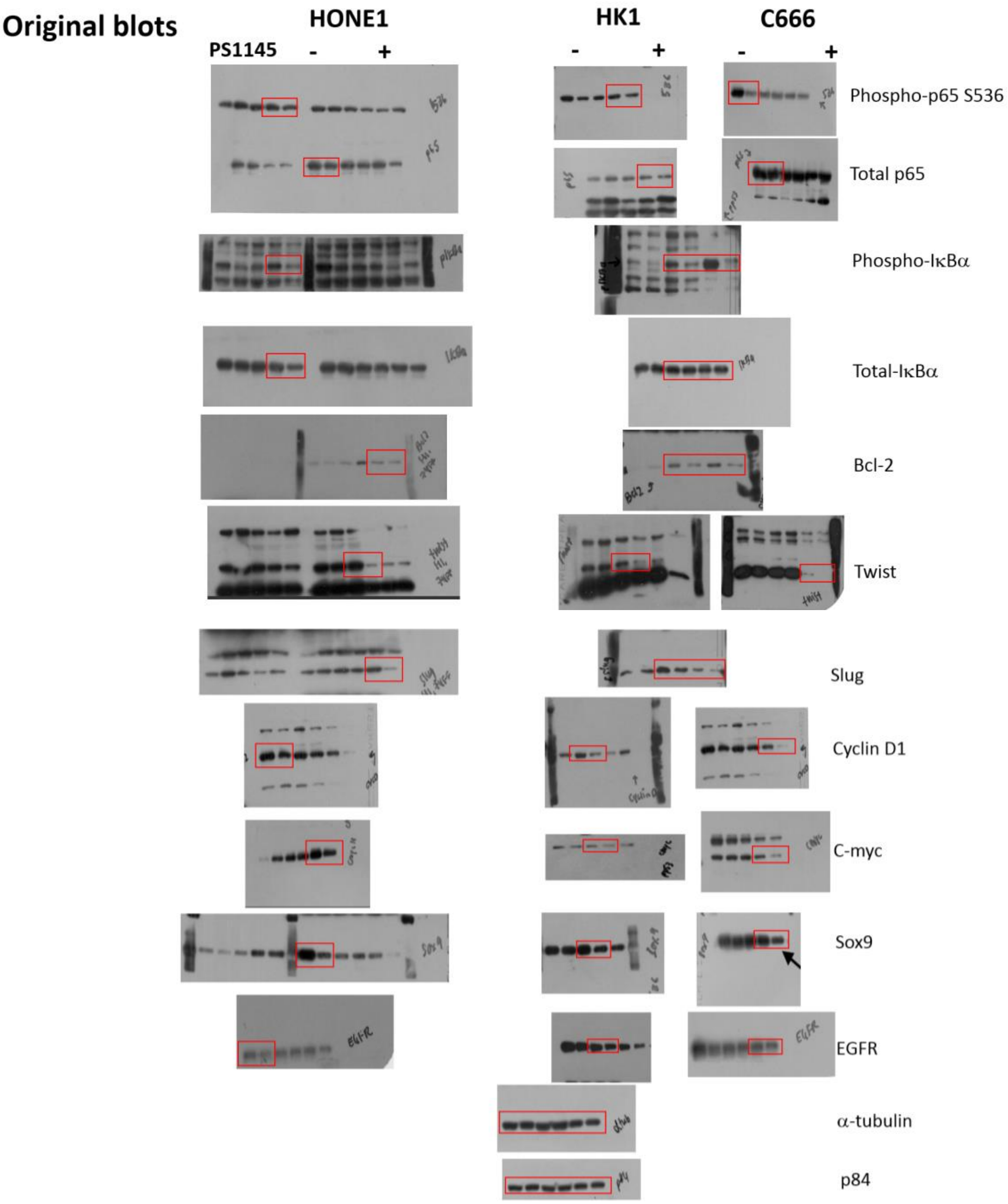

Figure S4. Full immunoblots for Fig. 3. Fig. 3 is shown on the upper panel and the original blots are shown on the lower panel. The red box indicated the cropped area in the original blot.

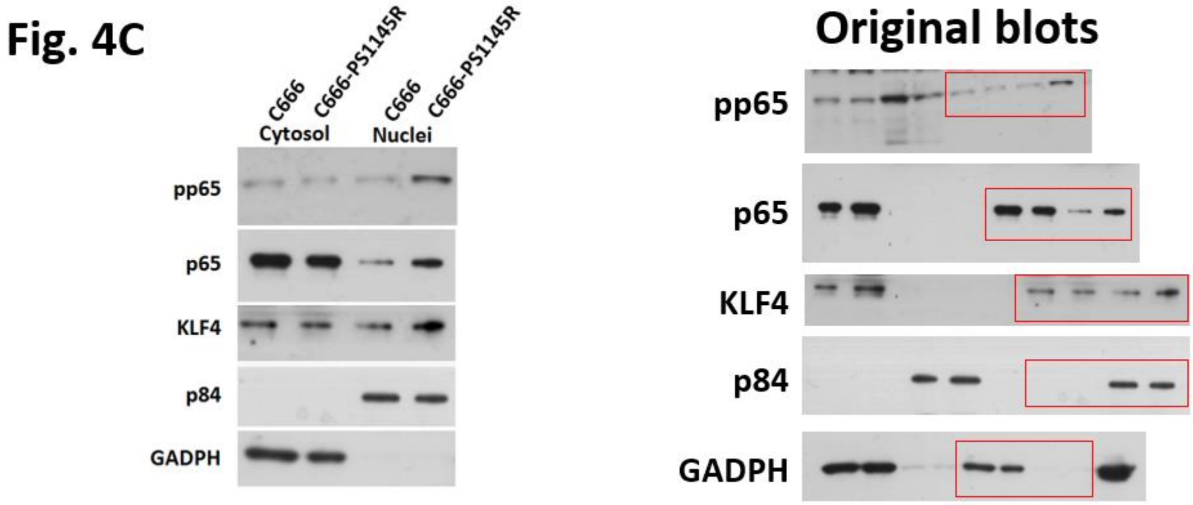

Figure S5. Full immunoblots for Fig. 4C. Fig. 4C is shown on the left and the original blots are shown on the right. The red box indicated the cropped area in the original blot.

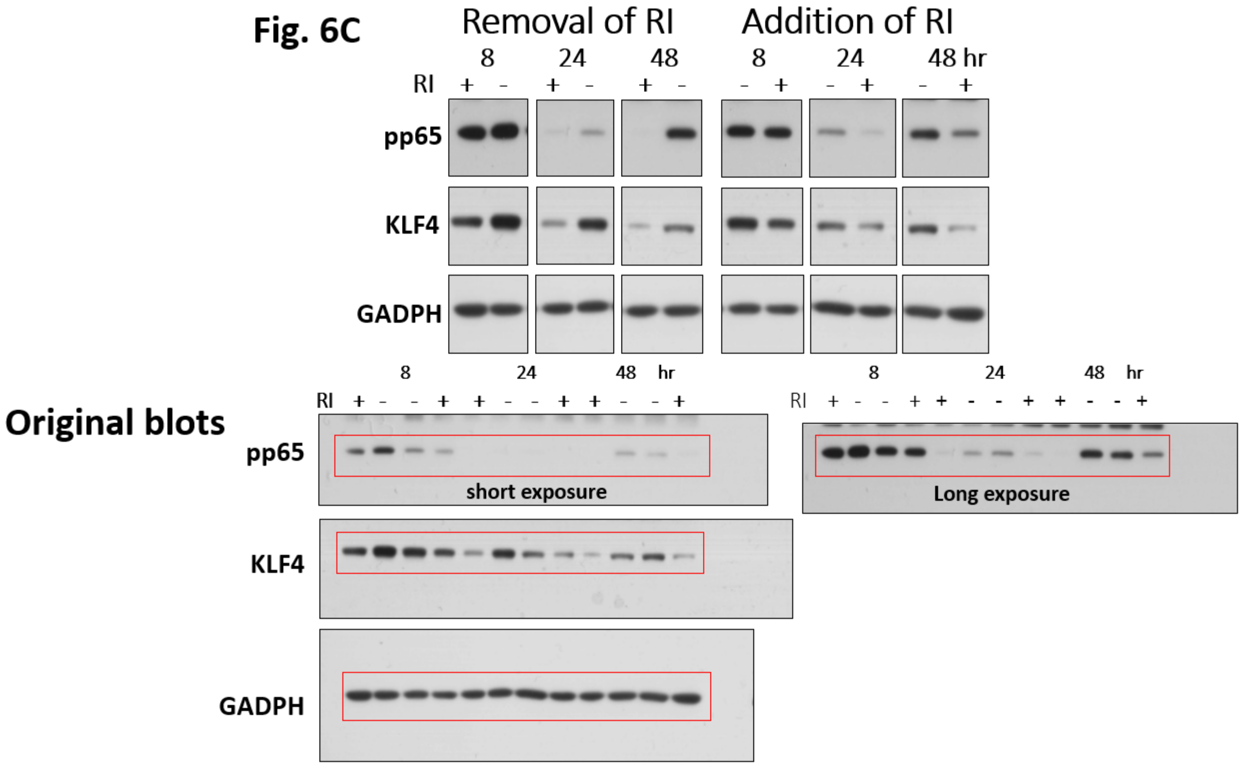

Figure S6. Full immunoblots for Fig. 6C. Fig. 6C is shown on the upper panel and the original blots are shown on the lower panel. Both long and short exposure versions for detection of pp65 are shown. The red box indicated the cropped area in the original blot.

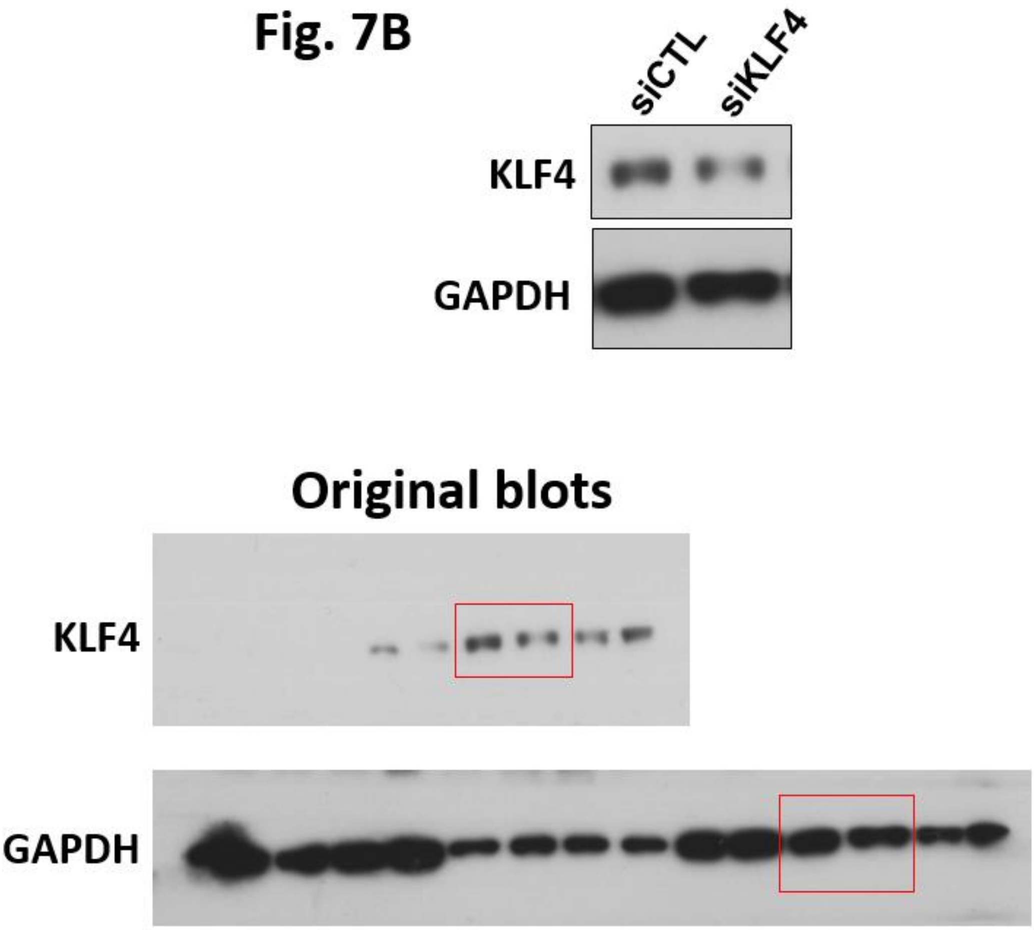

Figure S7. Full immunoblots for Fig. 7B. Fig. 7B is shown on the upper panel and the original blots are shown on the lower panel. The red box indicated the cropped area in the original blot.

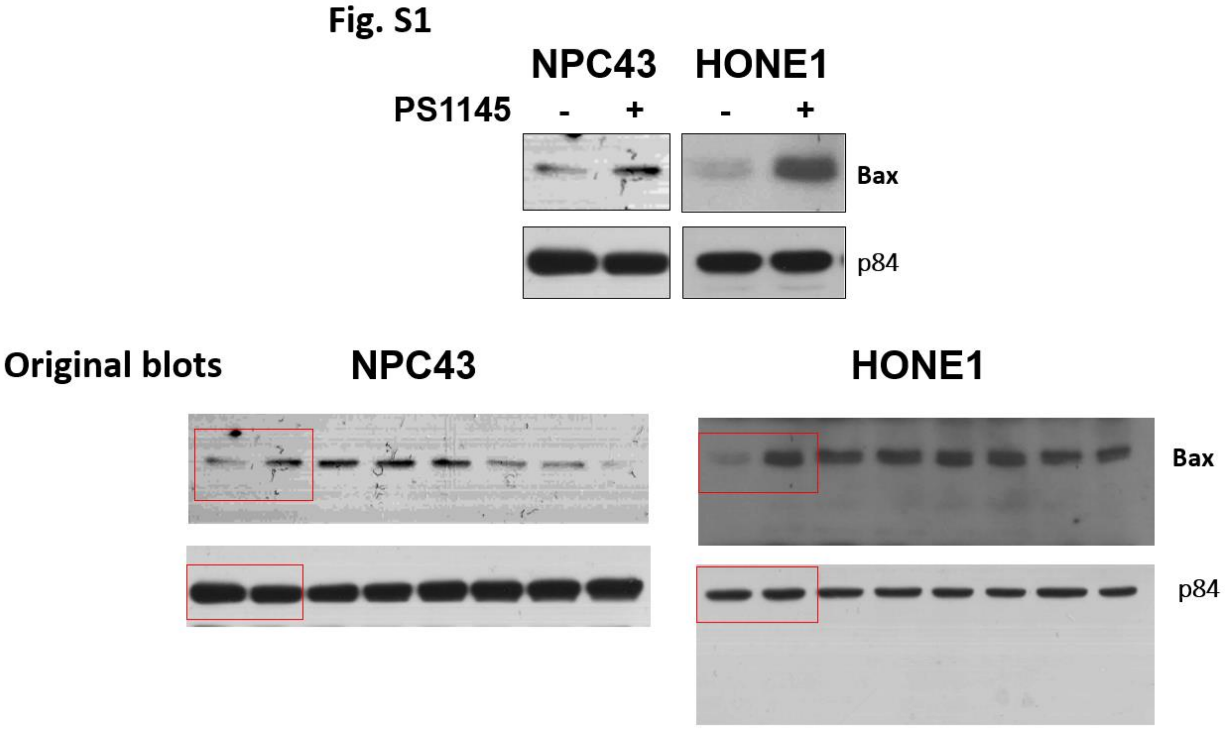

Figure S8. Full immunoblots for Fig. S1. Fig. S1 is shown on the upper panel and the original blots are shown on the lower panel. The red box indicated the cropped area in the original blot.

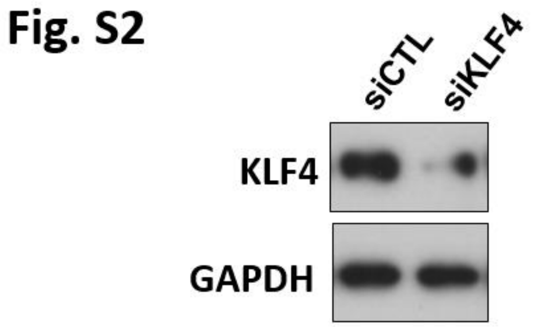

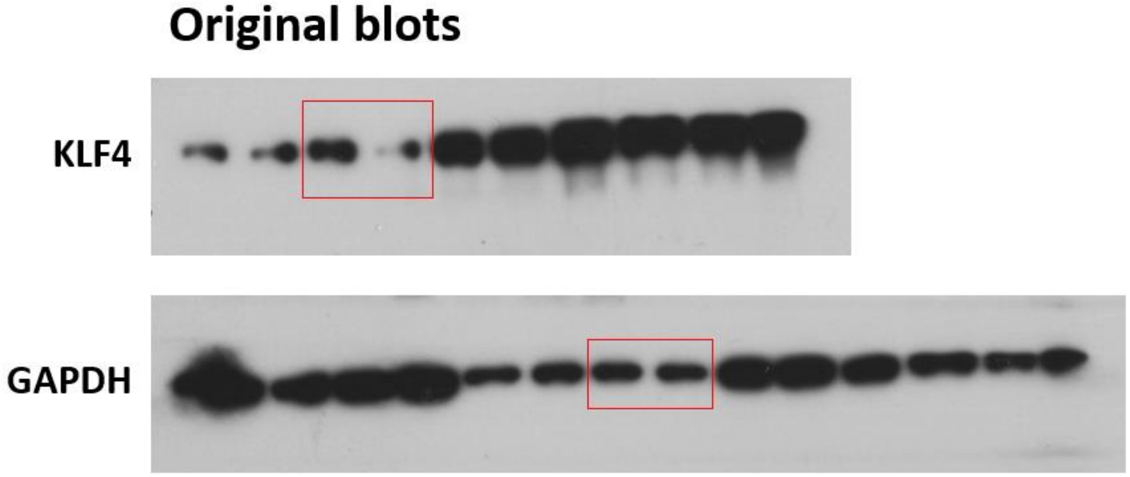

Figure S9. Full immunoblots for Fig. S2. Fig. S2 is shown on the upper panel and the original blots are shown on the lower panel. The red box indicated the cropped area in the original blot.

Table S1. Raw data of full measuremenmts of tumor sizes in the HONE1 solevnt control group (measured in mm)

	Length wk0	Wifth	Height	Length wk0.5	Wifth	Height	Length wk1	Wifth	Height	Length wk1.5	Wifth	Height	Length wk2	Wifth	Height
Animal															
1	0	0	0	10.8	6.1	1.8	0	0	0	0	0	0	0	0	0
2	0	0	0	0	0	0	8.5	5.8	1.7	16.3	8.3	5.1	14	13.3	6.5
3	0	0	0	10.8	7.8	0.5	14	8.6	3.5	11.7	13.9	5.5	20	9.8	6.1
4	0	0	0	7.5	7.6	1.9	11.5	9.4	5	8	3.7	2.5	20.4	15.5	7.5
5	0	0	0	8.8	7.5	3.6	14.6	6.3	1.9	10.5	5.2	6.4	17.2	13.7	7
6	0	0	0	10.1	9.2	2.9	4.6	7.6	3	14.3	10.9	2.7	13.5	10.6	5.2
7	0	0	0	0	0	0	16.8	8.9	2.8	14.7	7.4	4.4	21	12	5.2
8	0	0	0	11.3	5.7	1.4	13.6	9.7	3.6	18.4	10	7.9	18	9.8	4.6
9	0	0	0	16.1	8	1.9	11	9.3	2.9	8.3	6.5	5.4	20.4	12.4	6.5
10	0	0	0	9.7	6.9	0.5	11.1	9.3	2.3	18	9.1	4.6	21.8	14.3	6
11	0	0	0	0	0	0	10.7	9	5.5	20.7	10.7	4.6	22.1	12.1	4.5
12	12	5	0.5	14.1	6.5	1.5	15.4	7.5	2.3	17.6	14.2	5.7	14.7	12	5.5

Table S2. Averge tumor sizes in the HONE1 solevnt control group (measured in mm^3)

HONE1 solvent control - tumor size mm3

	week	0	0.5	1	1.5	2
animal	1	0	118.584	0	0	0
	2	0	0	83.81	689.979	1210.3
	3	0	42.12	421.4	894.465	1195.6
	4	0	108.3	540.5	74	2371.5
	5	0	237.6	174.762	349.44	1649.48
	6	0	269.468	104.88	420.849	744.12
	7	0	0	418.656	478.632	1310.4
	8	0	90.174	474.912	1453.6	811.44
	9	0	244.72	296.67	291.33	1644.24
	10	0	33.465	237.429	753.48	1870.44
	11	0	0	529.65	1018.854	1203.345
	12	30	137.475	265.65	1424.544	970.2
	average	2.727273	105.7565	322.5745	713.5612	1361.915
	SD	4.52267	51.86023	82.15622	225.9429	242.7365

Table S3. Raw data of body weights in the HONE1 solevnt control group (measured in g)

HONE1 solvent control

	wk	0	0.5	1	1.5	2
animal	1	14.9	16.3	14	15	16
	2	16.6	15.7	15.7	17.8	19.6
	3	13.9	14.6	13.9	17.9	18.2
	4	14.8	15.4	15.2	16.5	18.2
	5	16.3	14.9	17.3	17.2	17.5
	6	15.2	16.5	15	16	17.8
	7	15.6	13.8	14.9	18.6	16.7
	8	14.2	17.1	16.2	15.5	18.7
	9	13.7	15.5	15.2	16.6	17.2
	10	15.6	13.7	16.1	16	18.3
	11	16.4	15.6	16.8	15.8	16.2
	12	15.3	14.3	17.2	17.4	17.4
	average	15.20833333	15.28333	15.625	16.69167	17.65
	SD	0.479800306	0.53165	0.566539	0.547082	0.52158

Table S4. Raw data of full measuremenmts of tumor sizes in the HONE1 PS1145 treatment group (measured in mm)

	Length	Wifth	Height	Length	Wifth	Height	Length	Wifth	Height	Length	Wifth	Height	Length	Wifth	Height
Animal	wk0			wk0.5			wk1			wk1.5			wk2		
1	0	0	0	10.5	5.4	1.8	14.2	10.3	0.5	14.5	11.5	4	16.8	12.8	4.3
2	8	5	0.5	10.9	6.5	3	12	8.8	0.5	11.2	11.4	3.1	14	9.8	4.8
3	0	0	0	14.2	7.8	0.5	11.2	6.5	0.5	13.7	12.9	3	14.8	12.1	2.1
4	0	0	0	0	0	0	16.8	7.8	2.7	9.7	7.8	3	17.3	12	5.3
5	0	0	0	11.4	6.9	1.8	16.4	7.9	4	19.2	8.2	3	14.7	13.4	4.5
6	0	0	0	3.6	4.1	0.5	16.6	10.3	3	14.2	9	2.7	14.9	10.7	6.7
7	0	0	0	0	0	0	10.4	9.8	0.5	1.8	8.7	3.3	16.6	12.2	6.3
8	0	0	0	11.2	7.3	1.9	11.7	7.9	3.3	14.3	9.3	2.4	18.6	15.4	2.6
9	0	0	0	13	7	0.5	15.6	8.6	2.5	14	14.7	1.8	17.8	16	2.1
10	0	0	0	0	0	0	10.1	8.6	0.5	14.7	12	3	13.9	11.8	2.5
11	0	0	0	0	0	0	12.2	7.5	0.5	12.3	7.9	5.1	19.7	8.7	2.6
12	0	0	0	0	0	0	10.6	6.9	2.5	17.4	10	3.1	18.2	13.4	4.8

Table S5. Averge tumor sizes in the HONE1 PS1145 treatment group (measured in mm^3)

HONE1 + PS1145 - tumor size mm3

	week	0	0.5	1	1.5	2
animal	1	0	102.06	73.13	667	924.672
	2	20	212.55	52.8	395.808	658.56
	3	0	55.38	36.4	530.19	376.068
	4	0	0	353.808	226.98	1100.28
	5	0	141.588	518.24	472.32	886.41
	6	0	7.38	512.94	345.06	1068.181
	7	0	0	50.96	51.678	1275.876
	8	0	155.344	305.019	319.176	744.744
	9	0	45.5	335.4	370.44	598.08
	10	0	0	43.43	529.2	410.05
	11	0	0	45.75	495.567	445.614
	12	0	0	182.85	539.4	1170.624
	average	1.666667	59.9835	209.2273	411.9016	804.9299
	SD	2.886751	37.47365	93.72937	82.39778	155.9302

Table S6. Raw data of body weights in the HONE1 PS1145 treatment group (measured in g)

HONE1 + PS1145

	wk	0	0.5	1	1.5	2
animal	1	16.2	16.5	15.6	17.7	18.6
	2	15.7	16.4	16.4	18.1	19.2
	3	15.9	16.5	16.8	17.6	18.1
	4	17.4	15.9	16.9	17	18.7
	5	16.3	16	18	18.2	17.4
	6	15.3	15.7	17.2	17.3	17.6
	7	16.6	15	16.9	17.7	18.4
	8	16.3	18.1	16	16.5	17.4
	9	15.1	16.3	17.3	17.5	16.9
	10	17.7	15	18.6	16.8	19.2
	11	14.8	18.2	15.4	17.1	18.8
	12	16.1	16.4	16.6	18.5	17.4
	average	16.11666667	16.33333	16.80833	17.5	18.14167
	SD	0.429499356	0.498331	0.460957	0.296954	0.391651

Table. S7. Raw data of full measuremenmts of tumor sizes in the C666 solevnt control group (measured in mm)

	Length	Wifth	Height	Length	Wifth	Height	Length	Wifth	Height	Length	Wifth	Height	Length	Wifth	Height
Animal	wk0			wk0.5			wk1			wk1.5			wk2		
1	8	6	0.5	8	4	0.5	11.4	5.5	1.1	11.6	6.1	3.1	13.8	8.2	4.2
2	16	4	0.5	5	3	0.5	9.6	5.6	2.6	9.5	8.4	2.6	12.7	10.3	4.7
3	0	0	0	15	3	0.5	11.9	6	2.3	9.9	8.1	4.1	14.7	9.4	4.1
4	0	0	0	4	4	5	11.9	6.6	1.8	13	7.9	2.5	14.7	8	4.4
5	9	6	0.5	0	0	0	8.2	5.2	1.1	9	7	2.7	14.5	8.4	3.4
6	0	0	0	14	3	0.5	10.1	5.3	1.5	13	7.3	2.3	15.3	7.7	2
7	7	6	1	9	4	0.5	12.5	4.6	0.5	13.4	6.9	1.8	6.6	8.6	2.3
8	0	0	0	14	3	0.5	7.4	4.6	0.5	9	6.3	2.1	13.4	9.1	4.1
9	0	0	0	8	5	0.5	12.2	4.7	2.2	12.4	6	2.4	13	9.5	5
10	8	4	5	13	4	0.5	5.5	5	0.5	12.7	5.7	0.5	11.1	8.7	4.3
11	7	5	0.5	0	0	0	13.2	5.5	1.7	10.6	7.7	4	11.1	7	3.5

Table S8. Averge tumor sizes in the C666 solevnt control group (measured in mm^3)

C666 solvent control - tumor size mm3

	week	0	0.5	1	1.5	2
animal	1	24	16	68.97	219.356	475.272
	2	32	7.5	139.776	207.48	614.807
	3	0	22.5	164.22	328.779	566.538
	4	0	80	141.372	256.75	517.44
	5	27	0	46.904	170.1	414.12
	6	0	21	80.295	218.27	235.62
	7	42	18	28.75	166.428	130.548
	8	0	21	17.02	119.07	499.954
	9	0	20	126.148	178.56	617.5
	10	160	26	13.75	36.195	415.251
	11	17.5	0	123.42	326.48	271.95
	average	27.85	21.6	88.1655	200.8112	428.3728
	SD	24.53287	11.27793	28.79931	44.73609	83.89311

Table S9. Raw data of body weights in the C666 solevnt control group (measured in g)

C666 solvent control

	wk	0	0.5	1	1.5	2
animal	1	18.2	18.9	19.1	18.9	19.6
	2	20	18.4	19.8	18.9	18.5
	3	16.5	17.8	18.5	19.8	18.9
	4	19.4	16.7	18.2	17.9	19.5
	5	18.3	18.6	17.3	17.7	19.1
	6	20.8	16.8	18.6	18	18.1
	7	18.8	20.5	17	18.1	19.3
	8	19.2	18.5	18.8	18.6	19
	9	17.1	18.4	19.1	18.9	19.6
	10	19.2	19.2	18.2	17	19.7
	11	19.8	17.9	18.8	18.4	18.2
	average	18.84545455	18.33636	18.49091	18.38182	19.04545
	SD	0.630223626	0.533044	0.402153	0.377371	0.284924

Table S10. Raw data of full measuremenmts of tumor sizes in the C666 PS1145 treatment group (measured in mm)

	Length	Wifth	Height	Length	Wifth	Height	Length	Wifth	Height	Length	Wifth	Height	Length	Wifth	Height
Animal	wk0			wk0.5			wk1			wk1.5			wk2		
1	0	0	0	4	4	0.5	7.9	6	1.5	11.3	6.1	1.4	11.8	5.6	1.3
2	0	0	0	5	4	0.5	5.6	5.6	0.9	10.8	7	0.5	13	6.8	1.5
3	0	0	0	12	3	0.5	5	4.4	1.1	11.3	7.1	1.6	15.4	9.2	1.6
4	5	5	0.5	3	3	0.5	11.4	5.5	1.3	9.2	4	0.5	7.1	7.8	2.2
5	0	0	0	0	0	0	10.3	5.9	1.2	0	0	0	12.1	7.4	4
6	7	5	0.5	10	4	0.5	5	4.5	0.5	8.4	0.2	1.5	8.9	8.3	2.4
7	8	4	0.5	5	4	0.5	8.8	6.1	0.5	10.5	6.7	3.2	10.5	6.6	2.4
8	10	6	1	0	0	0	10.8	6.3	0.5	4.7	3.4	0.5	9.5	7	2
9	7	7	0.5	13	3	0.5	7.7	6.4	1.4	6	6	1.7	7.2	5.9	2
10	11	5	0.5	6	4	0.5	11	5	1.3	9.5	6.5	2.2	8.2	8.8	1.8
11	0	0	0	0	0	0	9.8	4.4	0.5	6.3	4.5	0.5	9.6	5.1	0.5
12	0	0	0	6	5	0.5	7.7	5.8	1.4	10.2	6.3	2.1	12.1	8.4	3

Table S11. Averge tumor sizes in the C666 PS1145 treatment group (measured in mm^3)

C666 + PS1145 - tumor size mm3

	week	0	0.5	1	1.5	2
animal	1	0	8	71.1	96.502	85.904
	2	0	10	28.224	37.8	132.6
	3	0	18	24.2	128.368	226.688
	4	12.5	4.5	81.51	18.4	121.836
	5	0	0	72.924	0	358.16
	6	17.5	20	11.25	2.52	177.288
	7	16	10	26.84	225.12	166.32
	8	60	0	34.02	7.99	133
	9	24.5	19.5	68.992	61.2	84.96
	10	27.5	12	71.5	135.85	129.888
	11	0	0	21.56	14.175	24.48
	12	0	15	62.524	134.946	304.92
	average	13.16667	9.75	47.887	71.90592	162.1703
	SD	9.038638	3.743934	12.73131	35.95264	47.27306

Table S12. Raw data of body weights in the C666 PS1145 treatment group (measured in g)

C666 + PS1145

	wk	0	0.5	1	1.5	2
animal	1	18.3	18.8	17.6	18.5	17.6
	2	19.1	18	19	18.4	19.6
	3	18.7	19.7	17.7	17.6	18.9
	4	18.6	17.2	18.2	16.8	18.7
	5	16	18.3	18.8	18.1	19.1
	6	18.4	18.5	17.8	17.9	17.9
	7	17.4	18.4	18.8	18.4	19.3
	8	18.1	18.3	18.1	17.3	19
	9	17.8	17.5	18.2	18.1	18.8
	10	18	19.5	19.5	18	18.1
	11	18.4	16.5	17.7	18	18.1
	12	19.2	17.4	18.2	17.8	18.4
	average	18.16666667	18.175	18.3	17.90833	18.625
	SD	0.42604595	0.464721	0.3	0.244446	0.303858
